# Association of liver function with health-related physical fitness: a cross-sectional study

**DOI:** 10.1186/s12889-023-16701-9

**Published:** 2023-09-15

**Authors:** Bo Ye, Jing Zhang, Zeyu Tan, Jiangang Chen, Xinliang Pan, Yuan Zhou, Wanwan Wang, Longlong Liu, Wenfei Zhu, Yuliang Sun, Ke Ning, Qian Xie, Ronghua Liu

**Affiliations:** 1 School of Physical Education, Shaanxi Normal University, Xi’an, China; 2 Department of Life Sciences, University of California Los Angeles, Los Angeles, USA; 3 School of Physical Education and Sports, Beijing Normal University, Beijing, China; 4 School of Kinesiology, Beijing Sport University, Beijing, China

**Keywords:** Liver function, Bilirubin, Body fat, Health-related physical fitness

## Abstract

**Background:**

In this study, by analyzing the correlation between various components of health-related physical fitness (HPF) and liver function indicators, the indicators of physical fitness that were highly correlated with liver function and could be monitored at home were screened to prevent more serious liver disease in the future, and to provide experimental basis for prescribing personalized exercise.

**Methods:**

A total of 330 faculties (female = 198) of a university were recruited. The indicators of HPF and liver function were measured. Spearman correlation analysis, multivariate linear regression, and cross-lagged panel model was used to data statistics.

**Results:**

In males, body fat (BF) was positively correlated with alanine aminotransferase (ALT); vital capacity and the vital capacity index were positively correlated with albumin; and vertical jump was positively correlated with globulin and negatively correlated with the albumin-globulin ratio (*P* < 0.05). However, there was no significant correlation among all indicators controlled confounding factors. In females, BF was negatively correlated with direct bilirubin; VO_2max_ was positively correlated with indirect bilirubin; and vertical jump was positively correlated with the albumin-globulin ratio and significantly negatively correlated with globulin (*P* < 0.05). Controlled confounding factors, body fat percentage was positively correlated with globulin (β = 0.174) and negatively correlated with direct bilirubin (β = –0.431), and VO_2max_ was positively correlated with indirect bilirubin (β = 0.238, *P* < 0.05). Cross-lagged panel analysis showed that BF percentage can negatively predict direct bilirubin levels with great significance (β = -0.055, *P *< 0.05).

**Conclusions:**

HPF may play a crucial role in liver function screening, particularly for female faculty members. For males, BF, vertical jump, vital capacity and vital capacity index could be associated with liver function but are susceptible to complex factors such as age, smoking, diabetes, and hypertension. In females, BF percentage is an important predictor of abnormal liver function in addition to VO_2max_ and vertical jump, which are not affected by complex factors.

## Background

With the development of scientific technology and society, there has been a significant change in the human disease chart: chronic noncommunicable diseases, including adiposis, hypertension, cancer, and cardiovascular diseases, have been substantial factors that affect human health. Therefore, the prevention of chronic diseases is indispensable for the well-being of citizens. Physical fitness is a biomarker of health [1]. The World Health Organization (WHO) defines health-related physical fitness (HPF) as follows: HPF is the ability for people to not feel too tired after dealing with daily work and to have the spare energy to enjoy leisure activities and deal with emergencies [2]. As a main approach to recent health evaluations, HPF is indicated by basic vital signs and motor functions of the human body, mainly consisting of body composition, cardiorespiratory endurance, muscular strength, balance, and flexibility. An increased risk of related chronic diseases is associated with deficits in HPF [3]. HPF represents sufficient energy to engage in daily activities as well as a relatively low risk of disease and is thus potentially associated with chronic disease prevention. According to relevant research, HPF can be a predictor of an array of chronic diseases, such as cardiovascular diseases, diabetes, and hypertension [4–6].

Since university faculty have a crucial role in national education, their health is interwoven with scientific and technological development and social progress [7]. Intensive work pressure, reduced physical activity, and long-term sedentariness have made faculty vulnerable to miscellaneous health problems. A study showed that there were 377 cases of abnormal liver function indicators(Alanine aminotransferase, Aspartate aminotransferase, Direct bilirubin, Indirect bilirubin, Albumin, Globulin or Albumin-globulin ratio is not within the normal range) based on physical examinations of a population of 2259 faculty members, accounting for 16.4% of the total [8]. A study has shown that the abnormal rate of liver function of some workers with different occupational exposure factors is 9.12% [9]. However, a study showed that the rate of abnormal liver function indicators of university faculty is 34.5% in a typical physical examination [10], which is higher than others. Although liver disease tends to develop silently with no signs or symptoms and there is little potential link between symptoms and liver disease, liver disease causes abnormal liver function indicators [11]. Therefore, by investigating the association between HPF indices and liver function indices, this study provides an experimental basis for the prevention of hepatic diseases, pertinent exercise interventions, and future exercise prescriptions for people who lack exercise and are sedentary.

## Methods

### Subjects

This study was conducted with 330 faculty members of universities in Shaanxi (198 females, 132 males). Among them, 85 participants in the sample (47 females and 38 males) were longitudinally tracked from 2018 to 2019. Excluding the individuals with body dysfunction, all subjects were capable of completing the test of HPF indices independently. All subjects were informed about the objective and content of the study and signed the informed consent form. The Ethics Committee of Shaanxi Normal University approved this study, and the ethical approval code is 202316007.

### HPF test

Based on the National Physique Determination Standards Manual and the actual circumstances [12], HPF testing indices were determined. The study adopted an InBody 230 Body Composition Analyser (InBody Co., Ltd., Seoul, Korea) to measure skeletal muscle mass, BF mass, and BF percentage; moreover, the skeletal muscle index was calculated. An HK6800-FH Electronic Spirometer (Shenzhen Hengkangjiaye Technology Co., Ltd., Shenzhen, China) was employed to measure vital capacity, and the vital capacity index was calculated. An Ergoselect 100 Cycle Ergometer (Ergoline GmbH Co., Ltd., Bitz, Germany) was applied with Astrand-Rhyming nomography to measure VO_2max_indirectly. A CAMRY Electronic Hand Dynamometer (Zhongshan Camry Electronic Co., Ltd., Zhongshan, China) was used to measure grip strength. Vertical jump was measured by a ZT-100 Vertical Jump Instrument (Yuejian Co., Ltd., Nantong, China). One-minute push-ups (for males)/sit-ups (for females) were counted with time measured by an HS-70W Stopwatch (Casio Computer Co., Ltd., Tokyo, Japan). The FUBO-TQ SAniR Instrument (Mengtuojia Co., Ltd., Yiwu, China) was adopted to measure sit-and-reach distances.

### Liver function test

After an 8–12-h fast, intravenous blood sampling was performed on each subject at the hospital using an AU480 Automatic Biochemical Analyser (Beckman Coulter Co., Ltd., Brea, CA, USA). The indices measured included alanine aminotransferase (ALT), aspartate aminotransferase (AST), direct bilirubin, indirect bilirubin, albumin, globulin, and the albumin-globulin ratio. Liver function indicators that are not within the normal range are described as abnormal liver function.

### Statistical analysis

The collected data were analyzed and processed using SPSS 23.0. The Kolmogorov‒Smirnov test was performed on the quantitative data: those data satisfying a normal distribution are represented by (Mean ± SD); the other data are represented by [M (IQR)].Because the data are not all normal distribution, the Mann‒Whitney U test was performed for intergroup comparisons, and Spearman rank correlation was used for bivariate correlation analysis. Based on correlation analysis, with confounding factors, including age, smoking, and chronic disease status, controlled multivariate linear regression (MLR) and the different multivariate models were implemented to analyze the correlation between HPF and biochemical indices. Moreover, the samples were longitudinally analyzed by a cross-lagged model based on the MLR. PASS 11.0 was adopted to calculate the sample size (*P* < 0.05, power > 0.80).

## Results

### Basic information

In the study, there were 132 male members with an average age of 43.59 ± 10.17 years, and average BMI of 24.84 ± 2.51 kg/m^2^; there were 198 female members with an average age of 40.24 ± 9.17 years, and average BMI of 22.10 ± 2.84 kg/m^2^. Among the male, 21 smokers (15.9%), 3 diabetic patients (2.3%), and 20 hypertensive patients (15.2%) were present; among the female, 3 diabetic patients (1.3%) and 8 hypertensive patients (3.3%) were present. The results indicated that the ALT level and indirect bilirubin level of male were significantly higher than those of female (*P* < 0.001); there were no significant differences in other liver function indices between male and female (Table 1).

**Table 1:** Test result comparison between male and female

	Male (*n* = 132)	Female (*n* = 198)	*P*
Age (years)	43.59 ± 10.17^a^	40.24 ± 9.17^a^	
Height (cm)	173.0 ± 55.41^a^	161.29 ± 9.99^a^	
Weight( kg)	74.47 ± 9.32^a^	57.89 ± 8.39^a^	
BMI (kg/m^2^)	24.84 ± 2.51^a^	22.10 ± 2.84^a^	
Smoking (percentage)	15.9%	0%	
Diabetes (percentage)	2.3%	1.3%	
Hypertension (percentage)	15.2%	3.3%	
ALT (U/L)	22.25 (18.20,28.63)^b^	15.55 (10.63, 22.98)^b^	< 0.001^**^
AST (U/L)	21.80 (18.45, 25.05)^b^	21.40 (15.15, 26.90)^b^	0.447
Direct bilirubin (µmol/L)	5.50 (4.70, 6.60)^b^	5.40 (4.60, 6.10)^b^	0.136
Indirect bilirubin (µmol/L)	11.53 ± 3.81^a^	7.91 ± 2.85^a^	< 0.001^**^
Albumin (g/L)	47.30 (45.60, 48.30)^b^	46.25 (45.00, 48.30)^b^	0.088
Globulin (g/L)	29.20 (27.80, 29.90)^b^	28.70 (27.70, 29.70)^b^	0.434
Albumin-globulin ratio	1.64 (1.54, 1.73)^b^	1.62 (1.54, 1.72)^b^	0.555

*BMI* body mass index, *ALT* alanine aminotransferase, *AST* aspartate aminotransferase. The normal range of indicator: ALT: 0 ~ 40.0 U/L; AST: 0 ~ 40.0 U/L; Direct bilirubin: 0 ~ 6.8 µmol/L; Indirect bilirubin: 3.4 ~ 17.0 µmol/L; Albumin: 35.0 ~ 55.0 g/L; Globulin: 20.0 ~ 30.0 g/L; Albumin-globulin ratio: 1.5 ~ 2.5

^**^Represents a correlation coefficient where *P* < 0.01

^a^Indicates that normally distributed data are expressed by ‘ ± s’

^b^Indicates that nonnormally distributed data are expressed by ‘median (quartiles)’

### Correlation between HPF and liver function indices

*According to the results, among male, there was a positive correlation between* BF mass and ALT (*r* = 0.217, *P* < 0.05); vital capacity and the vital capacity index were positively correlated with albumin (*r* = 0.243, *r* = 0.225, *P* < 0.05); and vertical jump was positively correlated with globulin and negatively correlated with the albumin-globulin ratio (*r* = 0.291, *r* = -0.260, *P* < 0.05) (Table 2). According to the results, among female, direct bilirubin was positively correlated with BF mass and BF percentage (*r *= -0.293, *r* = -0.347, *P* < 0.01); there was a positive correlation between VO_2max_ and indirect bilirubin (*r* = 0.212, *P* < 0.05); and vertical jump was negatively correlated with globulin and positively correlated with the albumin-globulin ratio (*r* = -0.189, *r* = 0.200, *P* < 0.01) (Table 3).Overall, body composition indicators are associated with ALT and AST, while strength indicators are associated with bilirubin indicators (Table 4).

**Table 2:** Correlation analysis between HPF and liver function indices among male (r-value)

	ALT	AST	Direct bilirubin	Indirect bilirubin	Albumin	Globulin	Albumin-globulin ratio
Skeletal muscle mass	0.046	0.010	0.052	− 0.086	0.099	− 0.022	0.045
Skeletal muscle index	0.007	− 0.003	0.078	− 0.019	− 0.028	− 0.103	0.095
BF mass	0.217*	− 0.034	− 0.088	− 0.123	− 0.034	− 0.056	− 0.024
BF %	0.194	− 0.026	− 0.115	− 0.097	0.092	− 0.028	− 0.050
Vital capacity	0.017	− 0.145	0.084	− 0.033	0.243*	− 0.007	0.173
Vital capacity index	− 0.050	− 0.121	0.105	0.034	0.225*	0.026	0.170
VO_2max_	0.085	0.018	0.130	− 0.030	− 0.045	− 0.109	0.098
Push-ups	0.094	− 0.061	− 0.053	− 0.101	0.144	− 0.010	0.089
Grip	− 0.090	− 0.004	0.072	0.024	0.075	− 0.101	0.058
Vertical jump	− 0.047	− 0.059	0.054	0.011	0.056	0.291*	− 0.260*
Sit-and-reach	− 0.160	0.001	0.074	0.020	− 0.097	− 0.055	0.048

*ALT* alanine aminotransferase, *AST* aspartate aminotransferase, *BF mass* body fat mass, *BF %* body fat percentage, *VO*_*2max*_ maximum oxygen uptake

^*^Represents a correlation coefficient where *P* < 0.05

**Table 3:** Correlation analysis between HPF and liver function indices among female (r-value)

	ALT	AST	Direct bilirubin	Indirect bilirubin	Albumin	Globulin	Albumin-globulin ratio
Skeletal muscle mass	0.145	0.047	0.040	0.118	− 0.085	0.017	− 0.041
Skeletal muscle index	0.124	0.096	0.057	0.057	− 0.095	0.053	− 0.06
BF mass	0.085	0.050	− 0.293**	− 0.036	0.068	0.162	− 0.097
BF %	0.050	0.038	− 0.347**	− 0.079	0.109	0.163	− 0.082
Vital capacity	0.009	− 0.027	0.017	0.003	− 0.008	− 0.034	− 0.084
Vital capacity index	− 0.086	− 0.049	0.107	− 0.016	0.002	− 0.101	0.119
VO_2max_	0.087	− 0.062	0.089	0.212*	0.072	0.122	− 0.027
Sit-ups	− 0.221	0.209	− 0.200	− 0.153	− 0.011	0.014	0.061
Grip	0.122	0.088	0.043	0.116	0.048	− 0.005	0.055
Vertical jump	0.054	− 0.049	0.091	0.107	0.063	− 0.189**	0.200**
Sit-and-reach	− 0.152	0.043	0.152	− 0.080	0.036	− 0.078	0.059

*ALT* alanine aminotransferase, *AST* aspartate aminotransferase, *BF mass* body fat mass, *BF %* body fat percentage, *VO*_*2max*_ maximum oxygen uptake

^*^Represents a correlation coefficient where *P* < 0.05

^**^Represents a correlation coefficient where *P* < 0.01

**Table 4:** Correlation analysis between HPF and liver function indices (r-value)

	ALT	AST	Direct bilirubin	Indirect bilirubin	Albumin	Globulin	Albumin-globulin ratio
Skeletal muscle mass	0.343**	0.033	0.601**	-0.245**	-0.150*	0.047	-0.001
Skeletal muscle index	0.324**	0.073	0.578**	0.254**	-0.163**	0.057	-0.014
BF mass	0.149**	0.031	-0.089	-0.091	0.083	0.117	-0.079
BF %	-0.076	0.018	-0.496**	0.086	0.117	0.076	-0.08
Vital capacity	-0.034	0.192**	0.416**	-0.186**	-0.166**	-0.007	0.108
Vital capacity index	-0.051	-0.034	0.145**	-0.027	-0.142*	-0.057	E
VO_2max_	-0.013	0.230**	0.339**	-0.056	-0.139*	0.060	0.031
Grip	0.033	0.306**	0.584**	-0.226**	-0.113	0.027	0.040
Vertical jump	-0.017	0.182**	0.345**	-0.103	0.062	-0.027	0.051
Sit-and-reach	0.011	-0.220**	-0.150	0.088	0.004	-0.076	0.029

*ALT* alanine aminotransferase, *AST* aspartate aminotransferase, *BF mass* body fat mass, *BF %* body fat percentage, *VO*_*2max*_ maximum oxygen uptake

^*^Represents a correlation coefficient where *P* < 0.05

^**^Represents a correlation coefficient where *P* < 0.01

### Regression analysis on HPF associated with liver function indices

By conducting correlation analysis, significantly correlated indicators of HPF and liver function were included as independent and dependent variables in the regression model. Age, smoking and chronic disease were set as control variables because they may affect liver function. In addition, because the correlation between independent variables is not strong, the model does not consider the collinearity and interaction between variables.

Model 1 included demographic variables, such as age, smoking, diabetes, and hypertension. Model 2 included demographic variables and HPF indices (BF mass, vital capacity index, vertical jump, sit-ups) related to dependent variables. According to the results, among male, there was no significant correlation between all HPF indices and each liver function index (*P* > 0.05) (Table 5). According to the results, among female, there was a significant positive correlation between BF percentage and globulin (β = 0.174, *P *< 0.05), a significant negative relationship between BF percentage and direct bilirubin (β = -0.431, *P* < 0.05), and a significant positive correlation between VO_2max_ and indirect bilirubin (β = 0.238, *P* < 0.05) (Table 6).

**Table 5:** Multivariant regression analysis of HPF indices associated with liver function index among male

		Model 1			Model 2		
Dependent variable	Independent variable	*β*	SE	*R*^2^	*β*	SE	*R*^2^
ALT	Age	− 0.089	0.085	0.054	− 0.126	0.088	0.104
	Smoking	− 0.015	2.143		0.031	2.109	
	Diabetes	− 0.149	5.650		− 0.123	5.585	
	Hypertension	0.200	2.425		0.232*	2.398	
	BF mass				0.169	0.179	
Albumin	Age	− 0.437*	0.018	0.238	− 0.434*	0.019	0.253
	Smoking	− 0.062	0.469		− 0.079	0.467	
	Diabetes	0.046	1.255		0.048	1.234	
	Hypertension	− 0.108	0.502		− 0.128	0.492	
	Vital capacity index				0.010	0.010	
Globulin	Age	− 0.152	0.020	0.050	− 0.234	0.024	0.127
	Smoking	0.015	0.509		0.044	0.623	
	Diabetes	− 0.083	1.303		0.039	1.810	
	Hypertension	− 0.095	0.559		− 0.004	0.648	
	Vertical jump				0.219	0.031	
Albumin-globulin ratio	Age	− 0.086	0.002	0.020	− 0.054	0.002	0.053
	Smoking	0.091	0.043		0.067	0.053	
	Diabetes	0.068	0.117		0.007	0.162	
	Hypertension	− 0.030	0.047		− 0.046	0.057	
	Vertical jump				− 0.220	0.003	

^*^Represents a regression coefficient where *P* < 0.05. Model 1: demographic variables; Model 2: demographic variables + HPF indices related to dependent variables. *ALT* alanine aminotransferase

**Table 6:** Multivariant regression analysis of HPF indices associated with liver function index among female

		Model 1			Model 2		
Dependent variable	Independent variable	*β*	SE	*R*^2^	*β*	SE	*R*^2^
ALT	Age	0.169*	0.068	0.036	0.104	0.101	0.089
	Diabetes	0.063	6.086		0.081	5.971	
	Hypertension	− 0.008	3.861		− 0.138	8.246	
	Sit-ups				− 0.190	0.069	
	Sit-and-reach				− 0.066	0.118	
AST	Age	0.234*	0.069	0.086	0.155	0.102	0.094
	Diabetes	− 0.118	6.099		− 0.149	5.960	
	Hypertension	0.103	3.568		0.094	8.222	
	Sit-ups				0.239	0.067	
Direct bilirubin	Age	0.001	0.009	0.007	− 0.026	0.013	0.242
	Diabetes	0.000	0.648		0.022	0.776	
	Hypertension	− 0.085	0.429		0.091	1.059	
	BFP				− 0.431*	0.021	
	Sit-ups				− 0.287	0.009	
Indirect bilirubin	Age	0.101	0.022	0.020	0.031	0.030	0.067
	Diabetes	− 0.014	1.690		− 0.066	2.128	
	Hypertension	0.125	1.119		− 0.120	1.391	
	Maximal oxygen				0.238*	0.524	
Globulin	Age	0.152*	0.021	0.036	0.202*	0.025	0.107
	Diabetes	− 0.077	1.579		− 0.118	1.566	
	Hypertension	0.081	1.045		0.052	1.235	
	BFP				0.174*	0.038	
	Vertical jump				− 0.076	0.025	
Albumin-globulin ratio	Age	− 0.222*	0.001	0.066	− 0.266*	0.002	0.107
	Diabetes	0.104	0.120		0.121	0.116	
	Hypertension	− 0.076	0.066		− 0.094	0.075	
	Vertical jump				0.060	0.002	

^*^Represents a regression coefficient where *P* < 0.05. Stepwise regression was implemented: Model 1: demography variables; Model 2: demographic variables + HPF indices related to dependent variables. *ALT* alanine aminotransferase, *AST* aspartate aminotransferase

### Cross-lagged panel analysis on HPF associated with liver function indices

**Fig. 1:**
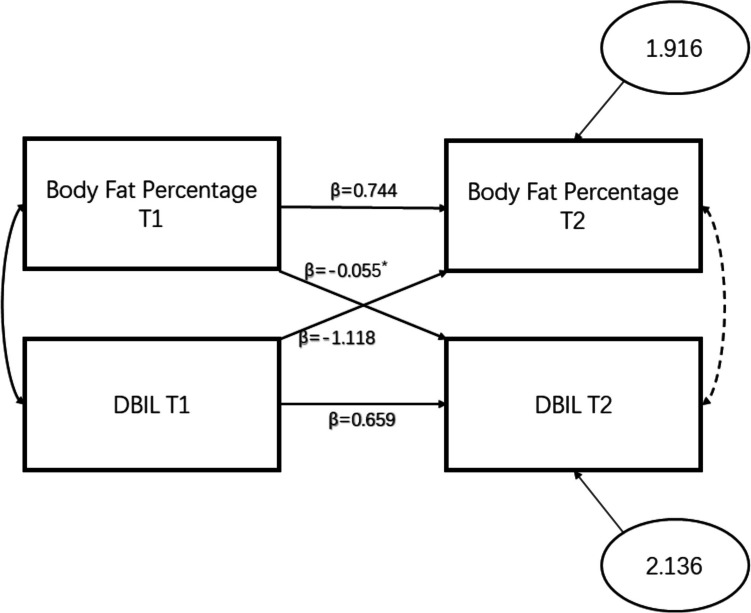
Cross-lagged panel

On a theoretical level, changes in healthy physical fitness may reflect liver function, and in turn, changes in liver function may also affect healthy physical fitness. Therefore, this study uses a cross lagged model to infer the direction of influence between variables. At the data level, some variables maintaining significant correlations over time are more appropriate to the use of cross lagged models. In this study, Body Fat percentage and Direct bilirubin were selected as variables for Cross-labeled Panel Analysis, with data from 2018 and 2019 included in the model while controlling for gender. The results was presented in Table 7 and Fig. 1. On the basis of BF% T1 and Direct bilirubin T1 correlation and BF% T2 and Direct bilirubin T2 correlation, BF% T1 and BF% T2 were stable correlated (SE = 0.063), Direct bilirubin T1 and Direct bilirubin T2 were stable correlated (SE = 0.153), BF% T1 and Direct bilirubin T2 were lagged correlated (SE = 0.026), and BF% T2 and Direct bilirubin T1 were lagged correlated (SE = 0.676) (Fig. 1).

**Table 7:** Correlation analysis between BF percentage and direct bilirubin (r value)

	Direct bilirubin T1	Direct bilirubin T2
BF % T1	− 0.467**	− 0.318**
BF % T2	− 0.309**	− 0.381**

^**^Represents a regression coefficient where *P* < 0.01; T1: first measurement in 2018; T2: second measurement in 2019. BF %: body fat percentage

According to the results, the BF percentage of in the first evaluation in 2018 (T1) and the BF percentage of in the second evaluation in 2019 (T2) were significantly negatively correlated with direct bilirubin in T1 and T2 (*r* = -0.467, *r* = -0.318, *r* = -0.309, *r* = -0.381, *P* < 0.01), which satisfied the condition for cross-lagged panel analysis (Table 7). According to the regression results, there was a significant positive correlation between BF percentage at T1 and BF percentage at T2 and between direct bilirubin at T1 and direct bilirubin at T2 (β = 0.774, β = 0.659, *P* < 0.01), which implied that BF percentage and direct bilirubin have relatively small variation with respect to time (Table 8). According to the cross-regression results, the correlation between direct bilirubin at T1 and BF percentage at T2 was not statistically significant; there was a significant negative correlation between BF percentage at T1 and direct bilirubin at T2 (β = -0.055, *P* < 0.05). Hence, BF percentage in T1 might have negative effects on direct bilirubin in T2 (Table 9).

**Table 8:** Multivariate linear autoregression of direct bilirubin associated with BF percentage

	BF% T2				Direct bilirubin T2		
	*β*	SE	Residual		*β*	SE	Residual
Sex	0.713	0.836	1.875	Sex	− 0.029	0.378	2.240
BF% T1	0.774**	0.063		Direct bilirubin T1	0.659**	0.153	

^**^Represents a regression coefficient where *P* < 0.01; T1: first measurement in 2018; T2: second measurement in 2019. *BF %* body fat percentage, *SE* error terms

**Table 9:** Multivariate linear cross regression of direct bilirubin associated with BF percentage

	Direct bilirubin T2				BF% T2		
	*β*	SE	Residual		*β*	SE	Residual
Sex	0.025	0.381	2.136	Sex	− 4.484	1.606	1.916
BF % T1	− 0.055*	0.026		Direct bilirubin T1	− 1.118	0.676	

Dependent variables: DBIL T2, BFP T2; * represents a regression coefficient where *P* < 0.05; T1: first measurement in 2018; T2: second measurement in 2019. *BF %* body fat percentage, *SE* error terms

## Discussion

According to the results of liver function tests in this study, the values were basically within the normal range for both males and females. In this study, BF mass for males was positively correlated with alanine aminotransferase (ALT); vital capacity and the vital capacity index were positively correlated with albumin; and vertical jump was positively correlated with globulin and negatively correlated with the albumin-globulin ratio. In females, BF mass and BF percentage were negatively correlated with direct bilirubin; VO_2max_ was positively correlated with indirect bilirubin; and vertical jump was positively correlated with the albumin-globulin ratio and significantly negatively correlated with globulin. After confounding factors were controlled, there was no significant correlation between HPF and liver function indicators in male members. In females, BF percentage was positively correlated with globulin and negatively correlated with direct bilirubin, and VO_2max_ was positively correlated with indirect bilirubin. Cross-lagged panel analysis showed that BF percentage can negatively predict direct bilirubin levels with great significance. In addition, there is a significant difference in ALT between males and females which was related to the fact that males usually have rich muscle because ALT is an important deaminase in protein metabolism.

The results of the study indicated that, for male, higher BF mass could mean higher ALT. A study showed that liver fat was correlated with ALT in children who were overweight and obese [13], which is similar to our study. The reason could be the results of metabolic adaptation to obesity in vivo. An increase in fat might result in a build-up of ketone bodies, including acetone, acetoacetic acid and *β*-hydroxybutyric acid, which are metabolized further by the body to protect itself from acidosis. Acetone is present in fairly small amounts and is absorbed immediately after it is formed. Acetoacetic acid and *β*-hydroxybutyric acid are both converted to oxaloacetic acid, which accelerates the shuttling of acetyl-CoA into the Krebs cycle, resulting in the rapid conversion of pyruvate. Consequently, ALT is activated, which induces alanine in skeletal muscle to generate pyruvate by combined deamination. This process could fully provide theoretical support for the results of this study. In addition, some research may support the findings of this study indirectly. For example, research has shown that obesity could be a hazard of liver dysfunction: obesity can increase hepatic cancer risk by 1.5- to 4.0-fold [14]. A prospective study of 900,000 Americans 16 years ago showed that the hepatic cancer risk of males and females with obesity was 4.52 times and 1.68 times higher, respectively, than that of an average person [15]. Hence, male members with high BF mass were encouraged to pay attention to controlling BMI to prevent hepatic diseases in advance.

For male, higher albumin could mean a higher vital capacity. A study pointed out that significant loss of muscle mass was observed in elderly people with low albumin levels [16]. This finding is consistent with the results of this study, which could be the reason why the vital capacity is correlated with respiratory muscles, given that protein is the building block of muscle. Therefore, a higher vital capacity was attributed to higher albumin. The results in this study also indicated that the BF percentage of female and the vertical jump were significantly positively correlated with their globulin; after controlling for variables such as age, diabetes, and hypertension, the correlation was still significant. This result could be attributed to increasing secretion of inflammation-inducing adipocytokines under obesity and other immune responses, since globulin, a mixture of an array of proteins, is in a category of important blood albumen proteins related to immune responses [17]. Globulin plays an important role in immunity and inflammation [18]. In addition, people with higher vertical jumps may enjoy physical activity. A study pointed out that moderate acute elevations in IL-6 and IL-10, such as those provoked by exercise, exert direct anti-inflammatory effects [19]. This response can induce globulin elevation. However, the relationship between vertical jump and globulin in male is opposite to that in female. After controlling for variables, the results were affected by age and other NCDs, such as hypertension. An investigation of 4576 adults (Juan Wang, et al.) [20] pointed out that, compared to people with normal blood pressure, the liver dysfunction risk of people with blood pressure near the upper bound of the normal value increased by 1.099 times, and the liver dysfunction risk of those who were diagnosed with hypertension increased by 2.290 times. The mechanism might be related to insulin resistance, intestinal microflora, and blood pressure coordination by the kidney [21–23]. This result showed that the albumin level was significantly negatively correlated with age. The correlation between the vital capacity and albumin level could be affected by age. Research by Lehallier et al. [24] also showed that as age increased, the albumin level of males decreased 0.015 g/dL per year, while that of females decreased 0.012 g/dL per year, which was consistent with the results of this study. One study pointed out that the albumin function of patients with advanced cirrhosis significantly decreased [25]. Some studies have shown that low skeletal muscle mass is associated with nonalcoholic fatty liver disease (NAFLD) [26–28]. It follows that patients with liver disease should enhance strength or resistance training to promote protein synthesis, which could have an active role in liver disease recovery.

Bilirubin is generated from the breakdown of haem present in haemoglobin and myoglobin, which is divided into direct bilirubin and indirect bilirubin and is a sort of bile pigment; direct bilirubin is produced by a combination of indirect bilirubin and D-glucuronic acid. Many studies have shown that bilirubin plays an important role in protecting the liver by inhibiting fat production. A study showed that bilirubin can prevent hepatic steatosis [29]. However, some studies have shown that bilirubin levels might increase abnormally upon red blood cell (RBC) destruction or hepatocyte dysfunction, thus constituting a substantial liver function indicator. Our study discovered that direct bilirubin in female members was negatively correlated with BF mass and BF percentage, which implies that a lower BF mass and BF percentage indicate higher direct bilirubin levels. In accordance with relevant research, adipocyte expansion of Mus musculus with obesity was mitigated by increasing bilirubin levels; at the same time, the expression of mRNA coding for NAD(P)H oxidase components and inflammatory markers in adipose tissue, such as TNF-α and HMGB1, was restricted; this suggested that bilirubin could improve insulin resistance by modifying inflammation and mitigating oxidative stress [30]. Recent research has shown that bilirubin could be a protective factor in several diseases [31]. One study showed that direct bilirubin was negatively correlated with BF mass and BF percentage, which could be ascribed to the metabolism of bilirubin to inhibit triglyceride lipase through inhibition of adenylate cyclase activity of fat cell mechanism [32]. This is consistent with the results of this study. Researchers also discovered that bilirubin could restrict the expression of fat-inducing genes in hepatic and renal tissues, as well as proteins that are of substantial significance in triglyceride synthesis, such as LXR α, mSREBP-1, FAS, and SCD-1 [33]. Therefore, a high bilirubin level, within a certain range, could probably mitigate fat synthesis, which could be a potential mechanism behind the negative correlation between direct bilirubin and BF percentage. Recently, increasing evidence has proven that bilirubin is not only a liver function indicator but also a powerful signalling molecule that could protect against obesity and other metabolic diseases [34]. There was a similar discovery in the longitudinal analysis of this study: a BF percentage decrease could be a potential cause of a bilirubin level increase, which suggested that improvement in obesity and a BF percentage decrease might promote an increase in direct bilirubin and metabolic adjustments, thereby protecting liver function. In addition, hereditary hyperbilirubinaemias include Gilbert-Meulengracht, Crigler-Najjar, Dubin-Johnson, and Rotor syndrome, and except for Crigler-Najjar syndrome, other hereditary hyperbilirubinaemias require no therapy [35]. The failure and fibrosis of the liver are associated with anaemia, and high bilirubin during liver disease was identified as a critical trigger of suicidal erythrocyte death leading to anaemia [36].

This study also showed a positive correlation between VO_2max_ and indirect bilirubin. Relevant research indicated that low-intensity and high-load aerobic exercise at 50% VO_2max_ could significantly increase total bilirubin levels [37]. Hinds et al. [38] suggested that aerobic exercise could probably increase bilirubin levels by increasing the induction of the rate-limiting enzyme BVRA, which could form bilirubin, restricting the activity of the bilirubin-combining enzyme UGT1A1 in the liver and controlling the function of bilirubin-PPARα. This finding was similar to our study. VO_2max_ is an important index that reflects people’s aerobic exercise ability. However, it is unclear which form of bilirubin elicits hepatic protection.

Admittedly, the main limitation is that the study did not use better imaging and gold standard diagnostic techniques, and the markers used lacked clinical reliability. Additionally, the study was limited by the fact that the subjects were only university staff. However, university staff are typical representatives of sedentary people, so the significance of this study was that it provided help for self-screening and assessment liver function for sedentary people. HPF indicators are convenient to use to measure and evaluate liver function indicators. More importantly, the causal relationship between HPF and liver function will be explored through longitudinal studies in the future.

## Conclusions

HPF may play a crucial role in liver function screening, particularly for female faculty members. For males, BF, vertical jump, vital capacity and vital capacity index could be associated with liver function but are susceptible to complex factors such as age, smoking, diabetes, and hypertension. In females, BF percentage is an important predictor of abnormal liver function in addition to VO_2max_ and vertical jump, which are not affected by complex factors.

## Data Availability

All data and materials are readily available.The datasets utilized and/or analyzed in the present study can be obtained from the corresponding author upon reasonable request.
